# Salicylic Acid Enhances Heat Stress Resistance of *Pleurotus ostreatus* (Jacq.) P. Kumm through Metabolic Rearrangement

**DOI:** 10.3390/antiox11050968

**Published:** 2022-05-13

**Authors:** Yan-Ru Hu, Yue Wang, Yu-Jie Chen, Qian-Qian Chai, Hao-Zhe Dong, Jin-Wen Shen, Yuan-Cheng Qi, Feng-Qin Wang, Qing Wen

**Affiliations:** Key Laboratory of Agricultural Microbial Enzyme Engineering, Ministry of Agriculture, Rural Department, College of Life Sciences, Henan Agricultural University, Zhengzhou 450002, China; yrhu@henau.edu.cn (Y.-R.H.); wangyue@henau.edu.cn (Y.W.); yujiec@henau.edu.cn (Y.-J.C.); chaixixi@henau.edu.cn (Q.-Q.C.); donghaozhe@henau.edu.cn (H.-Z.D.); shenjinwen@henau.edu.cn (J.-W.S.); qiyuancheng@henau.edu.cn (Y.-C.Q.); w_fengqin@henau.edu.cn (F.-Q.W.)

**Keywords:** heat stress, salicylic acid, *Pleurotus ostreatus*

## Abstract

*Pleurotus ostreatus* (Jacq.) P. Kumm is cultivated worldwide, and its growth is seriously threatened by heat stress. Here, we performed a comprehensive analysis to investigate the influence of the phytohormone salicylic acid (SA) in *P. ostreatus* under HS. The results showed that the hyphal growth recovery rate and the antioxidant capacity of *P. ostreatus* increased with exogenous SA application (0.01 mmol/L and 0.05 mmol/L) after HS treatment. Metabolomic and transcriptomic analyses showed that SA application (0.05 mmol/L) weakened central carbon metabolism to allow cells to survive HS efficiently. In addition, SA shifted glycolysis to one-carbon metabolism to produce ROS scavengers (GSH and NADPH) and reduced ROS production by altering mitochondrial metabolism. SA also maintained nucleotide homeostasis, led to membrane lipid remodeling, activated the MAPK pathway, and promoted the synthesis of cell-wall components. This study provides a reference for further study of SA in microorganisms.

## 1. Introduction

Heat stress (HS) is a major environmental stressor, and it is detrimental both to natural habitats [1] and agricultural production worldwide [2]. In plants, HS decreases the production and quality of crops by disrupting normal physiological processes like fertilization, pollen tube growth, and seed settling [2]. In the livestock industry, HS leads to economic losses due to slowed growth rates, reduced market weights, reduced fertility, and so on [1]. At the cellular level, organisms adapt to cellular stress by adjusting physiological/metabolic processes, changing gene expression [3], and altering chemical composition in response to different environmental conditions [4]. Under rising temperatures, evaluating the effect of heat on the crops is imperative to develop appropriate control measures. Hence, understanding the relationships between various adaptive responses and heat tolerance is valuable.

Salicylic acid (SA) is a common phenolic compound that regulates plant growth and responses to biotic and abiotic stresses. Particularly, it mediates local and systemic resistance against pathogen infection [5]. Genetic modification of endogenous SA levels affects plant resistance to pathogens [6]. In addition, endogenous SA protects plants from oxidative damage [7]. However, in *Ganoderma lucidum* (Curtis) P. Karst., exogenous SA inhibits complex III activity to generate reactive oxygen species (ROS) [8]. Thus, SA may play different roles in coping with oxidative stress in different species. SA also helps in coping with HS. Exogenous application of SA or acetylsalicylate has been shown to increase the thermotolerance of various plants, such as tobacco and *Arabidopsis* [9]. SA treatment affected energy absorption, utilization, and dissipation of excess energy in plants exposed to heat stress. In addition, SA decreased relative electrolyte leakage to increase their heat tolerance [5]. As a signaling molecule, SA has often been linked to oxidative responses and other pathways related to heat-stress responses, such as the calcium signaling pathway [9]. However, there is limited evidence of any such involvement in microorganisms.

*Pleurotus ostreatus* (Jacq.) P. Kumm is the second most cultivated edible mushroom in the world [10]. As a wood-rotting fungus, *P. ostreatus* can degrade various lignocellulosic substrates that are largely produced as by-products of agricultural, forestry, and food-processing activities [10]. As an edible mushroom, *P. ostreatus* has high nutritional and medicinal value. *P. ostreatus* is quite rich in protein, essential amino acids, and fiber other than a lower fat content [11]. In addition, *P. ostreatus* contains many bioactive components, such as polysaccharides and steroids [11]. Due to its high economic and ecological value, *P. ostreatus* has attracted a significant amount of research attention. *P. ostreatus* cultivation is strongly affected by seasonal temperature changes, especially HS. HS inhibits the growth rate of mycelia, increases apoptotic-like cell death, and decreases the resistance of *P. ostreatus* to pathogens, such as *Trichoderma asperellum* [12], thereby causing enormous economic losses. A few studies have addressed the resistance mechanism of *P. ostreatus* to heat stress. Signal transduction pathways are the key components of heat stress responses in fungi. Heat stress led to the excessive production of ROS, such as superoxide anion (O^2−^), hydroxyl radical (OH), and hydrogen peroxide (H_2_O_2_) in the mycelium and oxidative damage in *P. ostreatus*. At normal levels, the cellular ROS functions as a second messenger. However, the abnormal ROS accumulation can provoke damage to organisms by oxidizing macromolecules such as DNA, lipids, and proteins [13]. Signaling molecules such as nitric oxide (NO) could reduce the content of H_2_O_2_ by regulating mitochondrial aconitase [13]. In addition, metabolic changes were found to regulate heat stress resistance in *P. ostreatus*. Under heat stress, key metabolism changed significantly in *P. ostreatus* [14,15]. Some chemicals such as trehalose could alleviate heat stress in *P. ostreatus* by affecting central carbon metabolism [14,15]. Metabolomics analysis of *P. ostreatus* revealed that the endogenous SA content increased significantly after heat stress, though its function remained unknown [15].

In this study, the physiology of *P. ostreatus* was monitored after HS treatment in the presence and absence of exogenous SA. Furthermore, the metabolomic and transcriptomic parameters were analyzed. This study aimed to understand the role of SA in mediating HS resistance in *P. ostreatus*. This would not only help in the cultivation of *P. ostreatus* but also improve our overall understanding of the functions of SA in fungi.

## 2. Materials and Methods

### 2.1. Biological Material and Culture Conditions

The dikaryotic *Pleurotus ostreatus* (Jacq.) P. Kumm strain New 831, which was obtained from the College of Life Science, Henan Agricultural University, was used as the material strain. An activated mycelial block (5 mm) was inoculated on the mushroom complete medium (in g/L: yeast extract 5, glucose 20, VB1 0.01, MgSO_4_·7H_2_O 0.5, KH_2_PO_4_ 1, Agar 20) at 25 °C.

### 2.2. Temperature Stress and SA Treatments

In this study, temperature and SA were considered as two factors that divided the treatments into four groups, including No HS (without high temperature/without SA), No HS + SA (without high temperature/with SA), HS (high temperature/without SA), and HS + SA (high temperature/with SA) groups. Salicylic acid (Sigma-Aldrich, St. Louis, MO, USA) was dissolved in absolute ethanol to make a 1 mol/L stock solution, and then this was added to the melted mushroom complete medium at final concentrations of 0, 0.005, 0.01, 0.05, 0.1, 0.5, 1, and 2 mmol/L after filtration sterilization.

HS treatments were conducted as described previously [15]. An activated mycelial block (5 mm) was inoculated on the mushroom complete medium at 25 °C for 5 d and then at 40 °C for 24 h. To analyse the enzyme activity and the contents of H_2_O_2_, malondialdehyde (MDA) and proline, samples were collected by transferring the mycelia in sterilized plastic tubes, and they were frozen in liquid nitrogen immediately after heat treatment with three replicates per group. Then, the samples were stored at −70 °C until use.

### 2.3. Enzyme Activity Assays

To explore the mechanism by which SA affects ROS in *P. ostreatus*, we assessed the activities of five major antioxidant enzymes superoxide dismutase (SOD), catalase (CAT), glutathione reductases (GR), peroxidase (POD), and ascorbate peroxidase (APX) [16] using corresponding activity assay kits (Nanjing Jiancheng Bioengineering Institute, Nanjing, China: Superoxide Dismutase assay kit (WST-1 method), A001-3-2; Catalase assay kit (visible light), A007-1-1; glutathione reductases assay kit, A062-1-1; Peroxidase assay kit, A084-3-1; Ascorbate peroxidase test kit, A123-1-1). The enzyme activities were defined according to Ren et al., (2017) [16].

### 2.4. Detection of H_2_O_2_ and MDA Production

A previous study found that reducing the H_2_O_2_ content improves the tolerance of *P. ostreatus* to HS [13]. In addition, endogenous salicylic acid protects plants from oxidative damage [7]. In addition, the malondialdehyde (MDA) is a common assay for oxidative damage to membranes [17]. So, H_2_O_2_ and MDA content were analyzed: For this determination, an aliquot (0.50 g) of fresh mycelia was used from each sample. ROS production was measured using a reactive oxygen species assay kit (Nanjing Jiancheng Bioengineering Institute, Nanjing, China: E004-1-1). MDA production was measured using a malondialdehyde assay kit (TBA method) (Nanjing Jiancheng Bioengineering Institute, Nanjing, China: A003-1-2). The content of ROS was presented as the H_2_O_2_ in mmol/mg protein, and the content of MDA was calculated in nmol/mg of protein.

### 2.5. Detection of GSH and Proline Production

Because glutathione (GSH) and proline are two major ROS scavengers under HS [18], their accumulation in mycelia was analyzed. The same as in Section 2.4, fresh mycelial samples were used here. GSH production was measured using a reduced glutathione (GSH) assay kit (Nanjing Jiancheng Bioengineering Institute, Nanjing, China: A006-2-1). The proline production was measured according to a protocol described previously [19]. The contents of GSH in mg/g protein and of proline in µg/g fresh mycelia were calculated.

### 2.6. Determination of the Recovery Growth Rate of Hyphae

Environmental factors affected the hyphal growth and viability, and recovered hyphal fragments were able to initiate growth [20]. To some extent, recovery growth reflected the ability of hyphae to resist environmental stress. In this analysis, the hyphae were exposed to 40 °C for 24 h. Then, the hyphae were allowed to recover at 25 °C until one group of hyphae fully covered the medium. The recovery growth distance of hyphae in each group was measured, and the recovery growth rate of mycelia was further calculated using the formula: recovery growth rate (cm/d) = recovery growth distance (cm)/recovery growth days (d). Each treatment consisted of at least three replicates.

### 2.7. Metabolome Analysis

The mycelial samples (each sample with three biological repeats) were extracted with methanol: acetonitrile (2:1, *v*/*v*) and shaken for 1 min. The samples were placed in ice water for ultrasonic lysis for 10 min and then deposited at −20 °C for 2 h. The lysates were placed into a centrifuge for centrifugation (1284× *g*, 4 °C, 30 min), and 300 µL of each sample were placed into a 96-well plate. The 96-well plate containing supernatant was drained in a freeze dryer. Then, 300 µL of methanol (10%) was added to the drained plate, mixed evenly with a multitube vortexer (set to 2500 Hz, 3 min), and placed in ice water for 10 min with ultrasound treatment. The plate was centrifuged at 30,000× *g* and 4 °C for 20 min, and 250 µL of supernatant was taken for LC/TOF–MS analysis.

LC/TOF–MS analysis was performed using a 2777C UPLC system (Waters, Manchester, UK) coupled with a Xevo G2-XS QTOF (Waters, Manchester, UK). There were at least six biological replicates for each treatment in this metabolomics study. Metabolomics data were deposited in the EMBL-EBI MetaboLights database (DOI: 10.1093/nar/gkz1019, PMID:31691833) with the identifier MTBLS3836. Principal component analysis (PCA) and orthogonal projections to latent structures discriminant analysis (OPLS-DA) were used to separate these metabolites between experimental groups. The complete dataset can be accessed in the link https://www.ebi.ac.uk/metabolights/MTBLS3836, accessed on 1 June 2022.

Cells produce energy and intermediate metabolites through glycolysis, the pentose phosphate pathway, the TCA cycle, and mitochondrial respiration. When an organism is under stress, these key metabolic pathways rearrange to help organisms adapt to environmental changes [21]. Based upon the results of metabolite analysis, the pathways of energy metabolism, central carbon metabolism, and nucleotide metabolism were estimated [15]. In addition, membrane lipids remodeling was also estimated since phosphoglycerolipids are the main components of the cell membrane bilayer, and some also play an important role in cell signaling [22].

### 2.8. Transcriptome Analysis and qRT-PCR Verification

For this analysis, the total RNA isolated from six biological repeats sample was used to synthesize the DNA libraries. MGISEQ-based RNA sequencing was performed commercially on the MGISEQ-2000RS platform. The clean reads were obtained by removing the reads containing low quality, joint contamination, and high content of unknown base N. Then, the clean reads were mapped to the *P. ostreatus* genome (PC15 v2.0) using HISAT. The clean reads were mapped to the genome reference using Bowtie2, and then the expression levels of genes were calculated using RSEM. Differential gene expression analysis among the samples was performed using DEGseq. Thresholds of fold change ≥2 and adjusted *p* ≤ 0.001 were applied to assess the significant differences in transcript levels. The sequencing data were deposited in the NCBI Sequence Read Archive (SRA) database under the accession number SUB10696145.

For the determination of gene expression, total RNA was extracted using the RNAiso™ Plus (Takara) according to the manufacturer’s protocol and then was reverse transcribed using a PrimeScript RT Reagent Kit with gDNA Eraser (TaKaRa, Dalian, China). Subsequently, a quantitative reverse-transcriptase polymerase chain reaction (RT-qPCR) assay was performed on the Roche Light Cycler 480 Real-Time PCR System (Roche, Penzberg, Germany) with actin as a native control according to the protocol of SYBR Green Master Mix (Takara, Dalian, China) [23]. All the primers used in this experiment were listed in Supporting Information Table S2. The 2^−ΔΔCT^ method was used to determine the relative expression levels of the genes [24]. Similar results were obtained for each of the three experiments.

### 2.9. Statistical Analysis

Statistical analyses were performed using GraphPad Prism 6.0 (GraphPad Software, Inc., San Diego, CA, USA). The significant differences between multiple samples were conducted by One Way Analysis of Variance (ANOVA) following Turkey’s test (SPSS, Version 26.0, IBM Corporation, New York, NY, USA), and those between two samples were conducted by one-way ANOVA using GraphPad Prism 6.0. Data are represented as the mean ± SD of at least three independent experiments. A *p*-value less than 0.05 was considered significant.

## 3. Results

### 3.1. Physiological and Biochemical Changes

**Figure 1:**
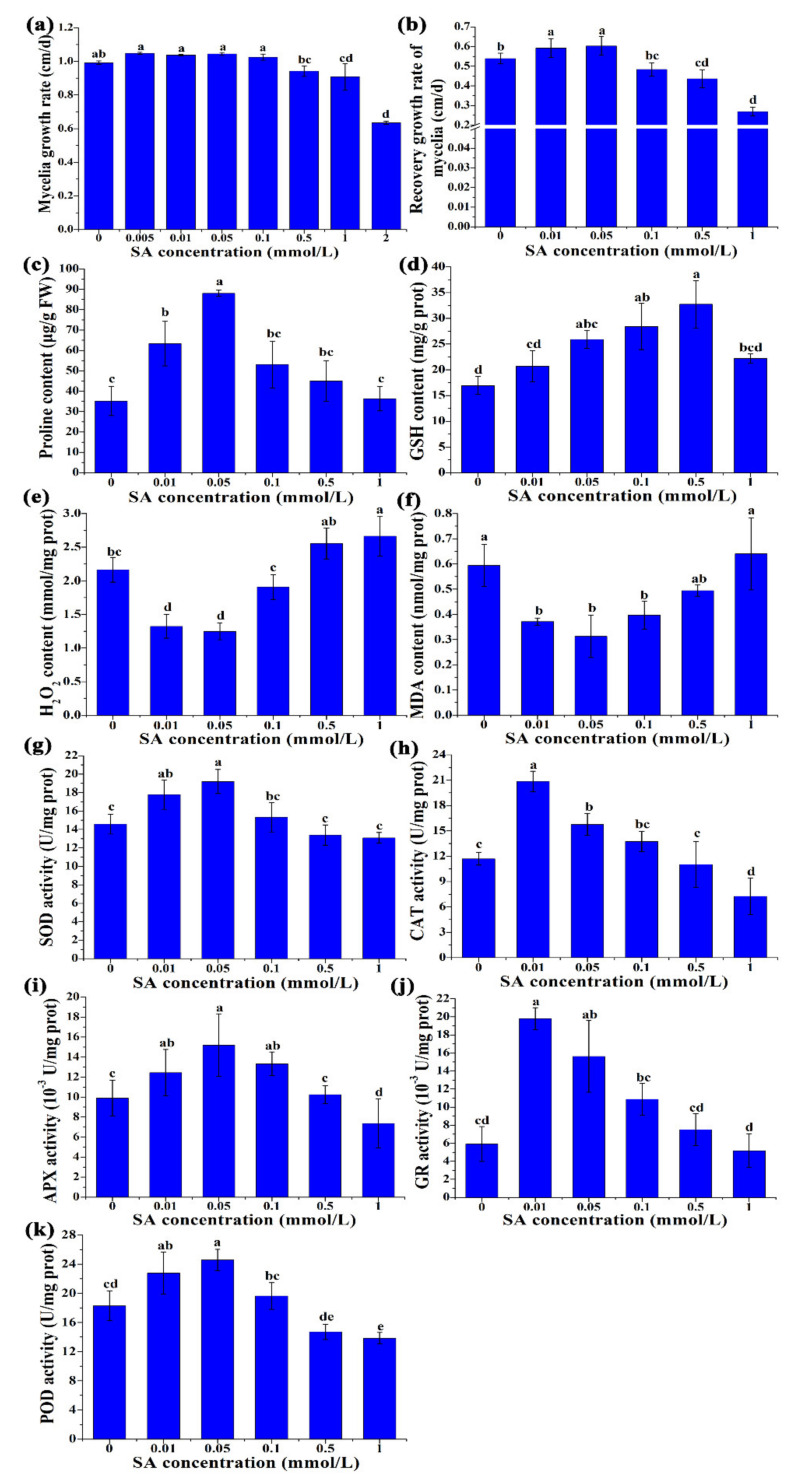
Physiological and biochemical changes with different concentrations of SA treatment under heat stress. (**a**) Mycelia growth rate; (**b**) recovery growth rate of mycelia; (**c**) proline content; (**d**) GSH content; (**e**) H_2_O_2_ content. (**f**) MDA content; (**g**) the activities of SOD; (**h**) the activities of CAT; (**i**) the activities of APX; (**j**) the activities of GR; (**k**) the activities of POD. The results are expressed as the means ± SD (*n* = 3). Different letters indicate significant differences between strains (*p* < 0.05, Tukey’s test).

The mycelial growth rates of *P. ostreatus* cultured on media with different concentrations of salicylic acid at 25 °C for 7 d are shown in Figure 1a. Treatment with 0.0050.5 mmol/L SA did not significantly change the mycelial growth rate. However, 1 mmol/L and 2 mmol/L SA significantly inhibited the mycelial growth rate (*p* < 0.05). Growth recovery experiments under heat stress showed that the 0.01 mmol/L and 0.05 mmol/L SA treatments significantly increased the recovery growth rate of mycelia (Figure 1b). Our study demonstrated that the content of H_2_O_2_ first decreased and then increased with increasing SA concentration. SA at concentrations of 0.01 mmol/L and 0.05 mmol/L significantly decreased the H_2_O_2_ content (approximately 57.6–61.2% of those in the control, *p* < 0.05). SA at concentrations of 0.1 mmol/L and 0.5 mmol/L had no significant influence on the H_2_O_2_ content (*p* > 0.05) compared with those in the control. However, 1 mmol/L SA significantly increased the content of H_2_O_2_ (approximately 1.2-fold of those in the control, *p* < 0.05) (Figure 1e). This result suggested that SA had a dose–concentration effect on ROS scavenging. The changes in MDA content showed that the 0.01 mmol/L and 0.05 mmol/L SA treatments caused a significant decrease (approximately 52.7–62.5% of those in the control, *p* < 0.05) in lipid peroxidation (Figure 1f).

To explore the mechanism by which SA affects ROS in *P. ostreatus*, we assessed the activities of five major antioxidant enzymes, CAT, SOD, APX, POD, and GR, and the accumulation of two major ROS scavengers, GSH and proline, under HS treatment (Figure 1). Compared with the control that did not add SA treatment, the proline and GSH contents significantly increased under 0.01 mmol/L and 0.05 mmol/L SA treatment (Figure 1c,d). On the other hand, SOD, POD, CAT, APX, and GR activity were significantly increased under the 0.01 mmol/L and 0.05 mmol/L SA treatments (*p* < 0.05) (Figure 1g–k).

All of the above results suggested that SA could enhance the resistance of *P. ostreatus* to heat stress, possibly by activating the functions of the antioxidant system.

### 3.2. Metabolite Changes

**Figure 2:**
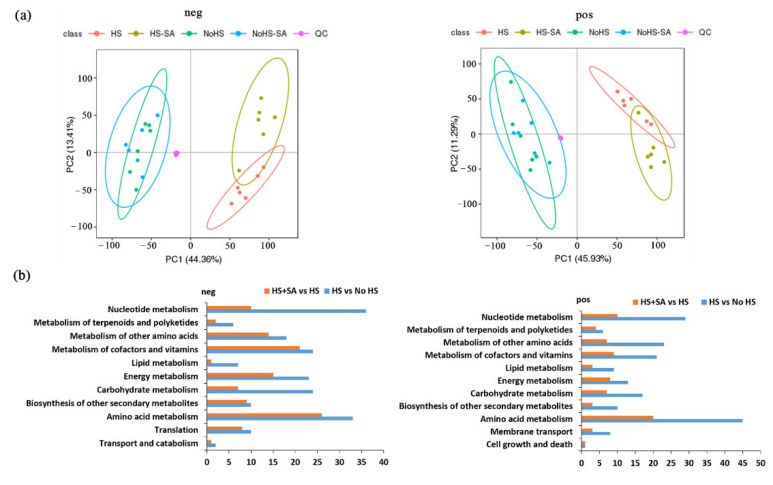
Metabolome and KEGG cluster analyses. (**a**) Principal component analysis (PCA) was used to separate metabolites between experimental groups. HS: heat stress; HS + SA: heat stress + salicylic acid; No HS: No heat stress; No HS + SA: No heat stress + salicylic acid; neg: the negative mode; pos: the positive mode; (**b**) KEGG cluster analysis of metabolites with significant differences.

To further investigate the mechanism by which SA enhances the resistance of *P. ostreatus* to heat stress, metabolomic analysis was performed. Differential metabolites were identified by a statistically significant analysis with *p* < 0.05 and VIP > 1. The results showed that each model was reliable for explaining the differences between the two groups and obtaining the different substances, and these data were available for subsequent of PCA and OPLS-DA analyses (Figure 2a and Table S1). In addition, metabolites were highly repeated between the No HS and No HS + SA groups that were clearly separated from the HS and HS + SA, while the metabolites of the HS and HS + SA treatments formed two separated groups (Figure 2a). These results indicated that HS greatly changed the metabolites of *P. ostreatus*, while SA mainly affected metabolism under HS.

The KEGG cluster analysis of metabolites (Figure 2b) further evidenced the effects of HS and SA on the metabolism of *P. ostreatus*. In the negative mode, 2114 metabolites changed significantly under HS compared with that in the No HS treatment, and 1160 metabolites changed significantly in HS + SA treatments in comparison with the HS group. In the positive mode, 1938 metabolites changed significantly under HS treatment in comparison with the No HS treatment, and 977 metabolites changed significantly in HS + SA treatment compared with the HS treatment. According to the KEGG cluster analysis, these differential metabolites belonged to “amino acid metabolism”, “metabolism of cofactors and vitamins”, “energy metabolism”, “metabolism of other amino acids”, “nucleotide metabolism”, “biosynthesis of other secondary metabolites”, “translation”, “carbohydrate metabolism”, “metabolism of terpenoids and polyketides”, “lipid metabolism”, “transport and catabolism”, “membrane transport,” and “cell growth and death” (Figure 2b). Comparing the HS + SA/HS group with the HS/No HS group, the major metabolite pathways with large differences in the quantity of differential metabolites were “nucleotide metabolism”, “carbohydrate metabolism”, “energy metabolism”, “lipid metabolism” and “amino acid metabolism”.

### 3.3. Energy Metabolism and Central Carbon Metabolism Analysis

**Figure 3:**
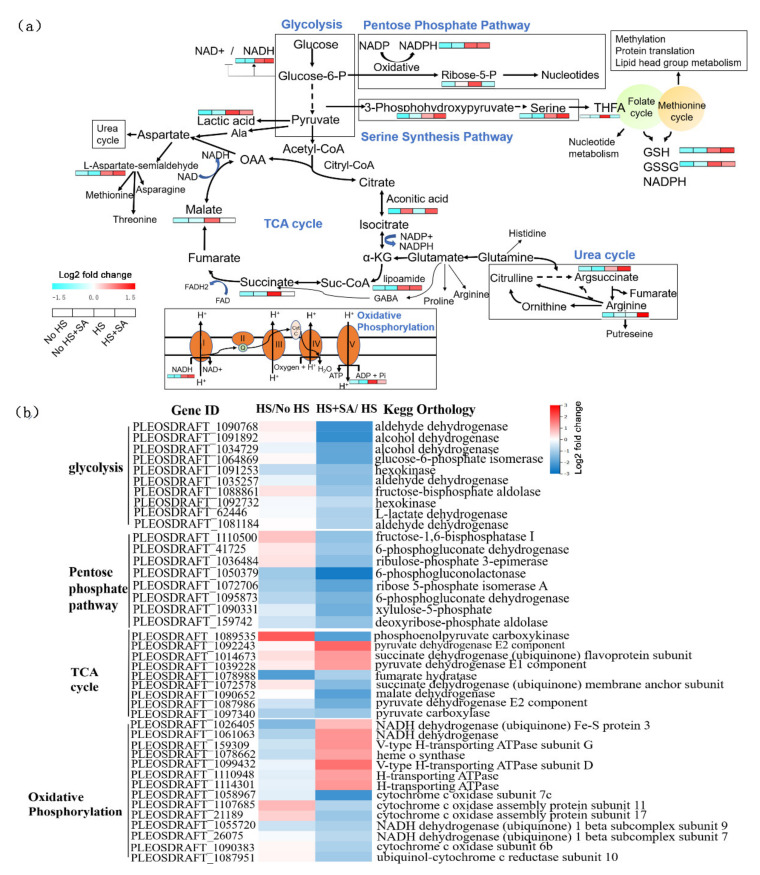
Energy metabolism and central carbon metabolism analyses. (**a**) Intermediate metabolite content analyse; (**b**) transcription level of related genes belonging to these metabolic pathways.

Our study showed that the intermediate metabolites in the PPP pathway (NADPH and ribose-5-P), serine synthesis pathway (3-phosphohydroxypyruvate and serine), folate-mediated one-carbon metabolism (tetrahydrogen folic acid, THFA), TCA cycle (malate, succinate, and lipoamide), oxidative phosphorylation (NADH and ADP), and glycolysis (lactic acid) significantly increased under HS treatment (Figure 3a). This suggested that the energy metabolism and central carbon metabolism of *P. ostreatus* may enhance under heat stress. However, the content of these metabolites decreased to different degrees under HS + SA treatment, except for NADPH, 3-phosphohydroxypyruvate, serine, lipoamide, and NADH. Interestingly, SA significantly increased the serine synthesis pathway and urea cycle under HS. In addition, SA significantly increased the GSH content and decreased the GSSG content (Figure 3a).

### 3.4. Nucleotide Metabolism Analysis

**Figure 4:**
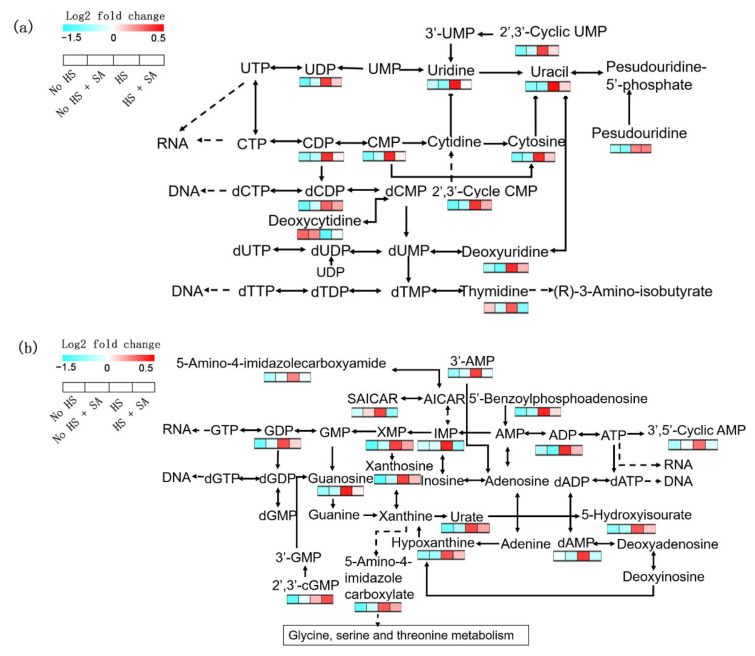
Nucleotide metabolism analysis. (**a**) Pyrimidine metabolism; (**b**) purine metabolism.

There were many changes in nucleotide metabolism with HS in *P. ostreatus*. Most of them are involved in pyrimidine and purine metabolism (Figure 4). In pyrimidine metabolism, the contents of UDP, uridine, 2′,3′-cyclic UMP, uracil, CDP, CMP, cytosine, pseudouridine, dCDP, 2′,3′-cyclic CMP, deoxycytidine, deoxyuridine, and thymidine significantly increased under HS treatment (Figure 4a). In purine metabolism, the contents of 5-amino-4-imidazolecarboxyamide, 3′-AMP, SAICAR, 5′-benzoylphosphoadenosine, GDP, XMP, IMP, ADP, 3′,5′-cyclic AMP, xanthosine, guanosine, urate, 5-hydroxyisourate, hypoxanthine, dAMP, 2′,3′-cGMP, and 5-amino-4-imidazole carboxylate significantly increased under HS treatment (Figure 4b). This phenomenon may be related to DNA fragmentation in *P. ostreatus* under HS treatment [25]. SA treatment significantly decreased the contents of nucleotide metabolism (Figure 4). This result suggested that SA may have a protective effect on nucleotides under heat stress.

### 3.5. Membrane Lipids Remodeling

**Figure 5:**
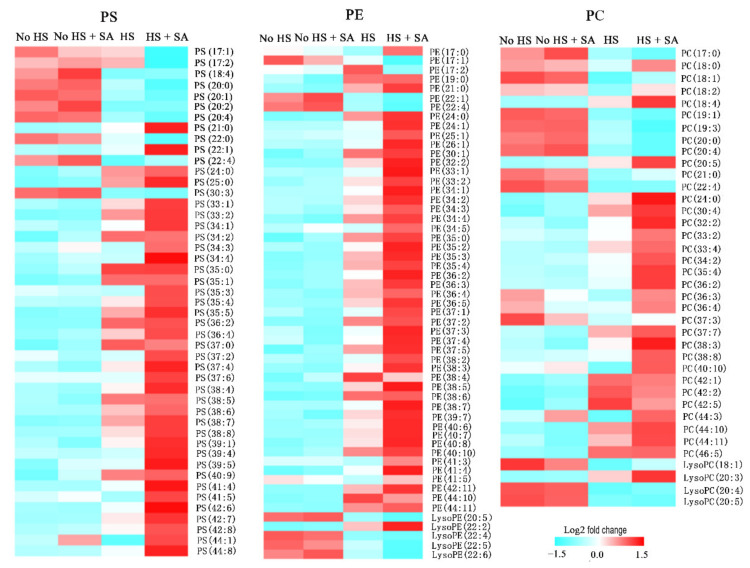
Phosphoglycerolipids composition analysis. PS: phosphatidylserine; PE: phosphatidylethanolamine; PC: phosphatidylcholine.

Lipid metabolism significantly changed in *P. ostreatus* under HS treatment. Our results showed that the contents of phosphatidylserine (PS), phosphatidylethanolamine (PE), and phosphatidylcholine (PC) species, especially long-chain unsaturated fatty acids, significantly increased under HS treatment. HS + SA treatment significantly increased these lipids further (Figure 5). However, the contents of phosphatidic acid (PA), phosphatidylglycerol (PG), and phosphatidylinositol (PI) species significantly decreased under HS treatment, and HS + SA treatment did not significantly change these lipid contents (Figure S2 in Supplementary Material). In addition, lysoPE, lysoPC, and lysoPA showed a decreasing trend in both the HS vs. No HS group and the HS + SA vs. HS group, in addition to lysoPE (22:2) and lysoPC (20:3).

### 3.6. Transcriptome Responses

To gain insight into biological processes at the genetic level, we analyzed transcriptome responses under four kinds of treatment (no HS, no HS + SA, HS, and HS + SA). Compared with HS treatment, 2290 genes were significantly upregulated, and 1912 genes were significantly downregulated after HS + SA treatment (Figure S1a). KEGG pathway enrichment analysis showed that the top 20 metabolic pathways with significant changes (sorted by Q value from smallest to largest) were “meiosis”, “glycerolipid metabolism”, “biotin metabolism”, “nucleotide excision repair”, “fructose and mannose metabolism”, “aflatoxin biosynthesis”, “pentose phosphate pathway”, and “pyrimidine metabolism”. The major metabolic pathways with the highest number of different genes were the “MAPK signaling pathway”, “carbon metabolism”, “glycerolipid metabolism” and “nucleotide excision repair and DNA replication” (Figure S1b).

“Carbon metabolism” analysis showed that HS + SA treatment significantly decreased the transcription levels of genes belonging to the glycolysis and pentose phosphate pathways compared with HS treatment alone. Nine genes in the TCA cycle significantly changed in HS + SA treatment compared with HS treatment alone, among which the transcription levels of three genes significantly increased and the transcription levels of six genes significantly decreased. This result suggested that SA addition weakened glycolysis, the pentose phosphate pathway, and the TCA cycle, which was consistent with the metabolic analysis results (Figure 3a,b). In addition, 14 genes in oxidative phosphorylation significantly changed under HS + SA treatment compared with HS treatment alone, among which transcription levels significantly increased in seven genes, and transcription levels significantly decreased in seven genes. The genes with increased levels of transcription under SA treatment mainly encoded proteins of complexes I (NADH dehydrogenase Fe-S protein 3 and NADH dehydrogenase) and V (v-type H-transporting ATPase subunit G and D and H-transporting ATPase), and the genes with decreased levels of transcription under SA treatment mainly encoded proteins of complexes III (ubiquinol-cytochrome c reductase subunit 10) and IV (cytochrome c oxidase subunit 7c and 6b and cytochrome c oxidase assembly protein subunit 11 and 17). This result was contrary to that for the HS vs. No HS group (Figure 3b).

To verify whether SA has a protective effect on nucleotides under heat stress, the transcription levels of genes belonging to “nucleotide excision repair and DNA replication” were analyzed. The result showed that 81 genes significantly changed. In the HS vs. No HS group, the expression levels of seven genes were significantly upregulated, and 74 genes were significantly downregulated under HS. This result suggested that HS weakened the repair function of nucleotides. In the HS + SA vs. HS group, the expression levels of 51 genes were significantly upregulated, and 30 genes were significantly downregulated under SA treatment (Figure S2). This result suggested that SA promoted nucleotide replication and repair to a certain extent. These results were consistent with those of nucleotide metabolism analysis.

To explain the metabolic changes of phosphoglycerolipids at the genetic level, we analyzed genes belonging to “glycerolipid metabolism”. The results showed that 42 genes significantly changed. In the HS vs. No HS group, the expression levels of nine genes were significantly upregulated, and 33 genes were significantly downregulated under HS. The nine transcriptionally upregulated genes were four speculative glycerol 2-dehydrogenase (NADP) (PLEOSDRAFT_52232, PLEOSDRAFT_1053140, PLEOSDRAFT_41480, and PLEOSDRAFT_28072), three speculative aldehyde dehydrogenases (NAD) (PLEOSDRAFT_1090768, PLEOSDRAFT_1041763, and PLEOSDRAFT_1081184), one speculative speculative alpha-galactosidase (PLEOSDRAFT_1043529), and one speculative phosphatidate phosphatase (PLEOSDRAFT_154122). In the “HS + SA vs. HS” group, the expression levels of 10 genes were significantly upregulated, and 32 genes were significantly downregulated under SA treatment. The 10 transcriptionally upregulated genes were three speculative phosphatidate phosphatases (PLEOSDRAFT_1033436, PLEOSDRAFT_1071023, and PLEOSDRAFT_1009219), two speculative lysophospholipid acyltransferases (PLEOSDRAFT_1040003 and PLEOSDRAFT_1064420), two speculative triacylglycerol lipases (PLEOSDRAFT_1028494 and PLEOSDRAFT_1058198), one speculative D-glycerate 3-kinase (PLEOSDRAFT_1040990), and one speculative alpha-galactosidase (PLEOSDRAFT_1083710) (Figure S3b).

**Figure 6:**
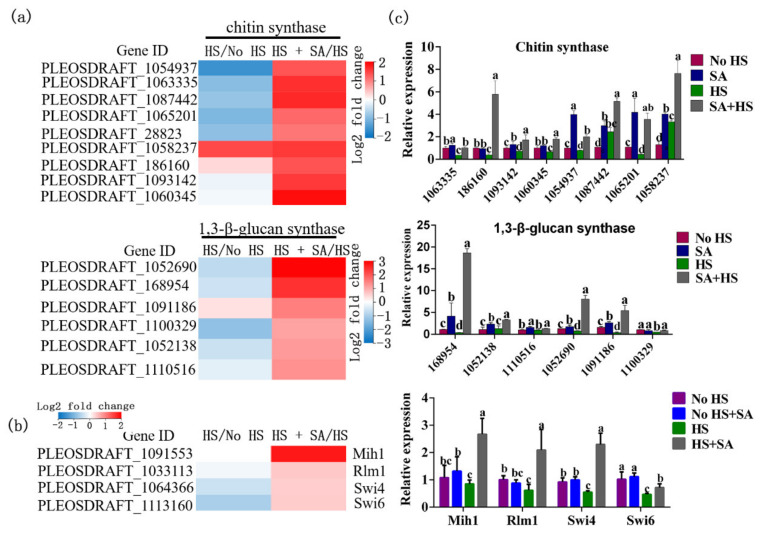
Gene expression analysis of the cell wall integrity pathway. (**a**) Changes in the expression of chitin and 1,3-β-glucan in the HS/No HS and HS + SA/HS groups; (**b**) the expression levels of major factors (Mih1, Rlm1, Swi4, and Swi6) in the “cell wall stress pathway”; (**c**) qRT-PCR results of cell wall integrity pathway genes. Different letters indicate significant differences between strains (*p* < 0.05, Tukey’s test).

Signal transduction is closely related to metabolism. In our results, of the 20 metabolic pathways with significant changes, the mitogen-activated protein kinase (MAPK) signaling pathway showed the greatest number of genes (267) with changes in expression. In the HS + SA vs. HS group, the expression levels of 203 genes were significantly upregulated, and 64 genes were significantly downregulated under SA treatment (Figure S4a). Seventy-three genes belonging to the “cell wall stress pathway” changed significantly. In the HS + SA vs. HS group, the expression levels of 51 genes were significantly upregulated, and 22 genes were significantly downregulated under SA treatment (Figure S4b). The expression levels of the major factors in cell wall integrity such as Mih1, Rlm1, Swi4, and Swi6 increased significantly under SA treatment (Figure 6b). Further analysis found that the expression levels of nine speculative chitin synthases and six speculative 1,3-β-glucan synthases increased significantly under SA treatment (Figure 6a). The qRT-PCR results were consistent with transcriptome results (Figure 6c). This result suggested that SA enhanced the synthesis of cell wall components by activating cell wall stress signals in the MAPK signaling pathway.

In addition to the MAPK signaling pathway, other signaling pathways, such as the Ca^2+^ signaling pathway, the heat shock protein signaling pathway, and the cAMP signaling pathway, were also reported to participate in the stress response and metabolic changes. Our results showed that HS activated the Ca^2+^ signaling pathway and that SA further enhanced it (Figure S5b). The genes that changed under SA treatment mainly belonged to the “Ca^2+^ pump” or “Ca^2+^ channel”. The content of cADPR, which functions in cellular signaling by regulating many Ca^2+^-permeable ion channels and evoking extracellular Ca^2+^, significantly increased under HS treatment and increased further under SA + HS treatment (Figure S5a). HS significantly increased the expression levels of two HSP20 family protein genes (PLEOSDRAFT_1090983 and PLEOSDRAFT_1090314) and one HSP70-interacting protein gene (PLEOSDRAFT_1076051). However, HS + SA treatment significantly decreased their expression levels and increased the expression level of one HSP20 family protein gene (PLEOSDRAFT_1094994) (Figure S5c). This result suggested that many HSPs played different roles in various environments. In addition, SA also activated the cAMP signaling pathway and the Hippo signaling pathway under HS (Figure S5d,e).

## 4. Discussion

We found that SA application alleviated ROS-mediated damages under HS by increasing the enzyme activities of the antioxidation system as well as the accumulation of GSH and proline (Figure 1). Metabolomic and transcriptomic analyses (Figure 2, Figure 3, Figure 4, Figure 5 and Figure 6) showed that SA application weakened the central carbon metabolism, enhanced serine metabolism or one-carbon metabolism, maintained nucleotide homeostasis, led to membrane lipid remodeling, activated the MAPK pathway, and synthesized cell wall components. The present study provides a reference for further research on the functions of SA in microorganisms.

HS threatens crop yields mainly through oxidative damage [17]. Hence, detoxification of ROS is crucial to cope with heat stress [26]. Organisms have developed ROS-scavenging systems consisting of antioxidant molecules (e.g., glutathione (GSH) and proline), and antioxidant enzymes (e.g., ascorbate peroxidase (APX), catalase (CAT), superoxide dismutase (SOD), guaiacol peroxidase (POD), and glutathione reductase (GR)) [18,26]. As an important component of signaling pathways in response to stress, SA is often linked to oxidative responses. It is used to increase the tolerance of crops to HS. For example, SA pretreatment could accelerate the restoration of photosynthetic function in plant leaves [5]. In alfalfa (*Medicago sativa* L.), SA pretreatment modulated the activities of APX, SOD, and POD; enhanced the ratios of reduced/oxidized homoglutathione (GSH); and alleviated oxidative stress triggered by cadmium [27]. Our findings suggest that SA helped *P. ostreatus* cope with ROS damage by regulating the activities of SOD, CAT, POD, APX, and GR and the accumulation of GSH and proline (Figure 1). In line with previous reports, our results demonstrated that SA is involved in various mechanisms of responses to multiple stressors depending on the stresses and/or the organisms.

In addition to oxidative damage, elevated temperature can also disrupt the balance in the cellular central energy metabolism of *Arabidopsis* [28], particularly the metabolic pathways of glycolysis, the TCA cycle, and oxidative phosphorylation. Our investigation indicated that intermediate metabolites (Figure 2, Figure 3 and Figure 4) in these major metabolic pathways and the expression levels of many related genes (Figure S1) were significantly increased under HS. The result suggested that central energy metabolism was activated under HS, which was consistent with a previous report [15]. SA addition weakened this change but maintained oxidative phosphorylation by increasing the expression levels of genes belonging to complexes I and V genes (Figure 3b). Elevated glycolysis and the TCA cycle might produce larger amounts of intermediate metabolites (such as NADPH, secondary metabolites, nucleotides, etc.) to help organisms adapt to ROS damage and maintain normal life activities [21]. However, glycolysis is a less efficient metabolic pathway compared to oxidative phosphorylation The metabolism of glucose to lactate during glycolysis generates only 2 ATPs per molecule of glucose, whereas oxidative phosphorylation generates up to 36 ATPs [29]. Increased metabolism consumes more nutrients and energy, which is not advantageous to growth recovery after stress relief. SA addition weakened this metabolism and maintained oxidative phosphorylation. This reduced energy consumption, allowed the cells to survive the heat stress efficiently, and had enough energy for rapid growth recovery after stress relief. This may explain why adding SA increased the recovery growth rate of *P. ostreatus* mycelia under HS (Figure 1b).

Interestingly, the pentose phosphate pathway was weakened, but the NADPH content did not significantly change with SA treatment under HS. This phenomenon led us to investigate other NADPH production pathways. We found that SA addition significantly enhanced the serine synthesis pathway under HS (Figure 3a). Intracellular serine is the major carbon source for folate-mediated one-carbon metabolism (1CMet), which operates in the cytosol and mitochondria to provide building blocks for S-adenosylmethionine (SAM), nucleotides, GSH, NAD(P)H, and ATP [30]. The content of tetrahydrofolate (THFA), a natural folate donor [31], was significantly decreased, while that of GSH was significantly increased with SA addition under HS. This suggested that GSH was produced via one-carbon metabolism through the folate cycle and methionine cycle. In addition, the folate cycle also mediates NADPH production [32]. This implied that SA shifted glycolysis to one-carbon metabolism. Therefore, most of the respondent metabolites and genes in glycolysis were concentrated downstream of glycolysis when SA was added under HS. However, the phenomenon of enhancement of one-carbon metabolism under SA treatment was rarely reported in fungi. The rewiring of metabolic programs during environmental adaptability has attracted much research interest, particularly in glycolysis and mitochondrial respiration [21]. We previously reported that a shift from mitochondrial respiration to glycolysis helped *G. lucidum* to cope with HS-induced ROS damage [21]. Yan et al., (2020) found that trehalose alleviated oxidative damage by enhancing PPP in *P. ostreatus* under heat stress [14].

Another interesting phenomenon was that pyruvate content was not significantly changed irrespective of the presence or absence of SA under HS. However, certain intermediate metabolites (such as aconitic acid, succinate, and malate) in the TCA cycle increased significantly under HS. So, where do those intermediate metabolites of the TCA cycle come from? When glycolysis is inhibited, alternative carbon sources such as glutamine and/or fatty acid have been shown to support TCA cycle upregulation [33]. Under stressful conditions, the non-essential amino acid glutamine is used by many cell types to fuel oxidative metabolism [33]. HS promotes glutamine metabolism in *G. lucidum*, according to previous research [21]. Further research is needed to determine the source of the carbon skeleton of the TCA cycle under HS and how heat stress activates remedial ways of the TCA cycle.

Because of their metabolic plasticity, mitochondria play a variety of roles in cellular metabolism, including controlling redox reactions, transcriptional regulation, and cell death [8]. According to our finding, HS treatment significantly increased related genes from mitochondrial complexes III and IV, while it significantly decreased related genes from mitochondrial complexes I and V. Under HS, the SA treatment produced the opposite result. Mitochondrial complex I connects the TCA cycle and oxidative phosphorylation to initiate mitochondrial respiration, and complex V functions to drive ATP synthesis [34]. Cytochrome c transfers electrons from complex III to complex IV, resulting in ROS production [35]. Although complex I can produce ROS, they only exist within the matrix. Complex III-generated ROS in the intermembrane space could escape into the cytosol [36]. Under HS, complexes I and V were damaged, while the expression levels of III and IV were enhanced, which increased the production of ROS and reduced energy production. SA addition ensured the smooth entry of substrates into the electron transport chain and ATP production by maintaining complexes I and V under HS. On the other hand, SA inhibited the transcription of III and IV genes to some extent to reduce ROS production. To deal with HS, ROS production should be reduced as much as possible to ensure the basic functions of the mitochondrial electron transport chain. This result also reflected the plasticity of mitochondrial metabolism.

As an important macromolecular substance in living organisms, lipid metabolism was altered significantly by SA treatment under HS. Remodeling took place in the components of phosphoglycerolipid, which was the main cell membrane lipid. The contents of PS, PE, and PC species, especially long-chain desaturation fatty acids, significantly increased under HS treatment. SA with HS treatment significantly increased these lipids further. However, the contents of PA, PG, and PI species were significantly decreased by HS treatment, whereas SA with HS treatment had no effect on these lipid contents. As the predominant scaffolds in the membrane, PC and PE account for more than half of the total phospholipid species in eukaryotic membranes. Their increase in content might replenish the membrane components for cell proliferation [37]. PS content may have increased in relation to signal transduction, as it is required for the targeting and function of several intracellular signaling proteins. In addition, PS functions in removing apoptotic cells [36]. PA is a signaling molecule that exhibits dynamic homeostasis under stress [36]. In our study, PA content decreased under HS. This could be because PA could bind NADPH oxidase and increase ROS production, which was harmful to cells under HS. In addition to phosphoglycerolipid species, the acyl chain length increased under HS and SA treatment. In response to environmental alterations such as temperature changes, the acyl chain gradually increases and then influences protein sorting and gene transcription [37].

Signal transduction and metabolism are inextricably linked. Our study found that SA activated various signaling pathways, such as the MAPK signaling pathway, the Ca^2+^ signaling pathway, the cAMP signaling pathway, and the Hippo signaling pathway, under HS. MAPK inhibitors decrease glycolytic activity and increase mitochondrial oxidative phosphorylation [38]. In addition, the MAPK signaling pathway serves a variety of physiological functions. In microbial cells, the cell wall is the first line of defense against environment changes. The cell wall of *P. ostreatus* has been reported to be damaged by heat stress [12]. In our result, the “cell wall stress pathway” in the MAPK signaling pathway was activated by SA. The expression levels of the major factors Mih1, Rlm1, Swi4, and Swi6 in the “cell wall stress pathway” [39] and the cell wall component synthetases (nine speculative chitin synthases and six speculative 1,3-β-glucan synthases) increased significantly under SA treatment (Figure 6). This result suggests that SA enhanced the synthesis of cell wall components by activating cell wall stress signals in the MAPK signaling pathway. Ca^2+^ is a versatile signaling molecule that regulates a wide variety of cellular metabolic pathways, such as the tricarboxylic acid (TCA) cycle, lipid metabolism reprogramming, and ROS homeostasis [40]. The cytosolic Ca^2+^ concentration is tightly regulated by an array of Ca^2+^ channels, transporters, exchangers, and pumps [32]. Our result showed that SA enhanced transcription levels of genes belonging to the “Ca^2+^ pump” or “Ca^2+^ channel”. The content of cADPR, which functions in cellular signaling by regulating many Ca^2+^-permeable ion channels and evoking extracellular Ca^2+^ entry [41], significantly increased under HS treatment and increased further under SA + HS treatment. These results showed that SA enhanced the Ca^2+^ signaling pathway by increasing the transcription levels of genes belonging to the “Ca^2+^ pump” or the “Ca^2+^ channel”. Another two signaling pathways, the cAMP signaling pathway and the Hippo signaling pathway, also play important roles in metabolism regulation [42]. These signaling pathways are often interrelated with each other [42]. In addition, heat-shock proteins (HSPs) and their cognates are primary mitigators of cell stress [43]. In our result, HS significantly increased the expression levels of HSP20 and HSP70 family protein, which was consistent with the previous findings [43]. However, SA with HS treatment decreased the expression levels of HSP20 and HSP70 family protein compared with only HS treatment (Figure S5c). According to Chen et al., (2018), HSPs proteins were expressed at basal low levels in the absence of stress, and significantly upregulated under heat shock and many other stress [43], so the decreased expression levels of HSPs in *P. ostreatus* under SA + HS treatment further suggested that heat damage in *P. ostreatus* under SA + HS could be significantly lower than that under HS treatment.

## 5. Conclusions

SA shifted glycolysis to one-carbon metabolism to generate ROS scavengers (GSH and NADPH). Furthermore, SA reduced ROS production by modifying mitochondrial metabolism. On the other hand, SA caused membrane lipid remodeling (long-chain desaturation PC, PE, and PS significantly increased) and enhanced the synthesis of cell wall components by activating cell wall stress signals in the MAPK signaling pathway. SA reduced ROS production by reprogramming metabolism, allowing cells to survive efficiently under HS conditions. In addition, SA activated several signaling pathways. More research is needed to determine whether SA affects metabolic processes directly by affecting metabolic genes or through signaling pathways. The regulation of SA on *P. ostreatus* adaptability under heat stress is complicated. We will investigate the molecular mechanism of SA alleviating heat stress in *P. ostreatus* in the future, such as the regulation mechanism of exogenous SA on one-carbon metabolism, central energy metabolism, signaling transduction, and so on. Furthermore, the relationship between SA and various signaling pathways is worth investigating.

## Data Availability

The complete metabolome dataset can be accessed here https://www.ebi.ac.uk/metabolights/MTBLS3836, accessed on 1 June 2022. The sequencing data have been deposited in the NCBI Sequence Read Archive (SRA) database under the accession number SUB10696145.

## Supplementary Materials

The following supporting information can be downloaded at: https://www.mdpi.com/article/10.3390/antiox11050968/s1, Table S1: OPLS-DA scatter plots of LC–MS data of *P. ostreatus* samples in each of the two groups; Table S2: Primers used for real-time RT-qPCR in this study; Figure S1: Transcriptome responses and KEGG pathway enrichment analysis; Figure S2: Heatmap of changes in gene transcription levels associated with nucleotide excision repair and DNA replication in the HS/No HS and HS + SA/HS groups; Figure S3: Heatmaps of phosphoglycerolipid composition and transcription changes of genes belonging to “glycerolipid metabolism”; Figure S4: Heatmaps of MAPK signaling pathway related genes; Figure S5: Other signaling pathway analyses.
