# Supplementary material for: Salicylic Acid Enhances Heat Stress Resistance of Pleurotus ostreatus (Jacq.) P. Kumm through Metabolic Rearrangement
Source: Antioxidants (Basel). 2022 May 13;11(5):968. doi: 10.3390/antiox11050968 (PMC9137821; doi:10.3390/antiox11050968)
Supplement: Supplementary file 1 [file antioxidants-11-00968-s001.zip › antioxidants-1710904-supplementary.pdf]

Supplementary Material for Antioxidants

Salicylic acid enhances heat stress resistance of *Pleurotus ostreatus* through metabolic rearrangement

Yanru Hu, Yue Wang, Yujie Chen, Qianqian Chai, Haozhe Dong, Jinwen Shen, Yuancheng Qi, Fengqin Wang, Qing Wen*

Affiliations: Key Laboratory of Agricultural Microbial Enzyme Engineering, Ministry of Agriculture, Rural Department, College of Life Sciences, Henan Agricultural University, Henan, Zhengzhou 450002, People's Republic of China.

Running title: Important role of salicylic acid in Pleurotus ostreatus under high temperature stress

**Corresponding author. Mailing address: College of Life Sciences, Henan Agricultural University, 95 Wenhua Road, Zhengzhou 450002, People's Republic of China. Telephone and Fax: 0371-63555790. E-mail: wenqing@henau.edu.cn*

Supplementary Material

Table. S1 OPLS-DA scatter plots of LC-MS data of *P. ostreatus* samples in each of the two groups

mode	group	R^2	Q^2	pvalue (R^2)	pvalue (Q^2)
pos	HS+SA/HS	0.9977	0.8135	0	0.01
pos	HS+SA/No HS+SA	0.9984	0.9409	0	0.005
pos	HS/No HS	0.9993	0.9646	0	0.005
pos	No HS+SA/No HS	0.9906	0.5836	0.03	0
neg	HS+SA/HS	0.9960	0.8243	0	0
neg	HS+SA/No HS+SA	0.9990	0.9178	0	0.005
neg	HS/No HS	0.9995	0.9733	0	0.01
neg	No HS+SA/No HS	0.9922	0.7837	0.005	0

R^2＞0.5 and Q^2＞0.5 indicated a good model.

Table. S2 Primers used for real-time RT-qPCR in this study

Target gene	primer
PLEOSDRAFT_1052690	F-RT-1052690：GGGGATTTGAAGAATAAGC
	R-RT-1052690：CAACCACATCAGCGAGTA
PLEOSDRAFT_168954	F-RT-168954：ACCTCTGTAGCAATCTCCC
	R-RT-168954：GTTTCGTGCCAAGTTCA
PLEOSDRAFT_1091186	F-RT-1091186：GGCAATCAACCACCAAG
	R-RT-1091186：TCGCTCAAGGAACCAGA
PLEOSDRAFT_1100329	F-RT-1100329：ACCGAAAGAGGACGATG
	R-RT-1100329: GCCAGTAAGTAGGTAAATAGG
PLEOSDRAFT_1052138	F-RT-1052138：CCGTGCTGGGTGTTATT
	R-RT-1052138：TCGGTTCGTCTCGTCAT
PLEOSDRAFT_1110516	F-RT-1110516：AACGGCACTAAGGATGA
	R-RT-1110516：CAAGAACCACAACACCAG
PLEOSDRAFT_1091553	F-RT-Mih1：ACCCGCCTCACATAACT
	R-RT-Mih1：CTGCCATCTTTCCCAAC
PLEOSDRAFT_1033113	F-RT-Rlm1：ACTGCTCAACCGACATCC
	R-RT-Rlm1：CTTCACCTTCCCATCCC
PLEOSDRAFT_1064366	F-RT- Swi4：CGCTGCTCCTAAACCTG
	R-RT- Swi4：GGCGTCATTGTTCCATC
PLEOSDRAFT_1113160	F-RT- Swi6：ACCACCGTCCACCTCCG
	R-RT- Swi6：ATCCGTGCGTCCAGTCC
PLEOSDRAFT_1054937	F-RT-1054937：CCGTCGTTGAAGATACCA
	R-RT-1054937：CGTTAGGCAAGCCGTTA
PLEOSDRAFT_1063335	F-RT-1063335：CTACTGCTGGCTACTTACCT
	R-RT-1063335：TGTTCCCTATTTCATTTCC
PLEOSDRAFT_1087442	F-RT-1087442：CGACCGATGTTTATGCTG
	R-RT-1087442：CGGAACTCAATGATGTAGAAG
PLEOSDRAFT_1065201	F-RT-1065201：ACCTTAATTCCTCATCTACCA
	R-RT-1065201：CCCATCTTTACCACTGTCC
PLEOSDRAFT_28823	F-RT-28823：GAGCATCTATCGCTTGTG

	R-RT-28823：CATGGACTCTGTCGTCTTA
PLEOSDRAFT_1058237	F-RT-1058237：TATCATTCTGTCGTTAGCC
	R-RT-1058237：TTTATTGTCTCCCTTGGT
PLEOSDRAFT_186160	F-RT-186160：TGAGTTTGGTGTAAGTTCG
	R-RT-186160：ATCGTCCCTGGGTATGG
PLEOSDRAFT_1093142	F-RT-1093142：GGGGCACTGAATAAGGC
	R-RT-1093142：TCCAGCGTAGTGATTGATA
PLEOSDRAFT_1060345	F-RT-1060345：ATTGGAAGGTCTTGCTC
	R-RT-1060345：TAACAGGTTGCGTGATG
actin	F-RT-ACT: GTGCTGTTTTCCCTTCAATCG
actin	R-RT-ACT: CTCCATATCGTCCCAGTTAGTG

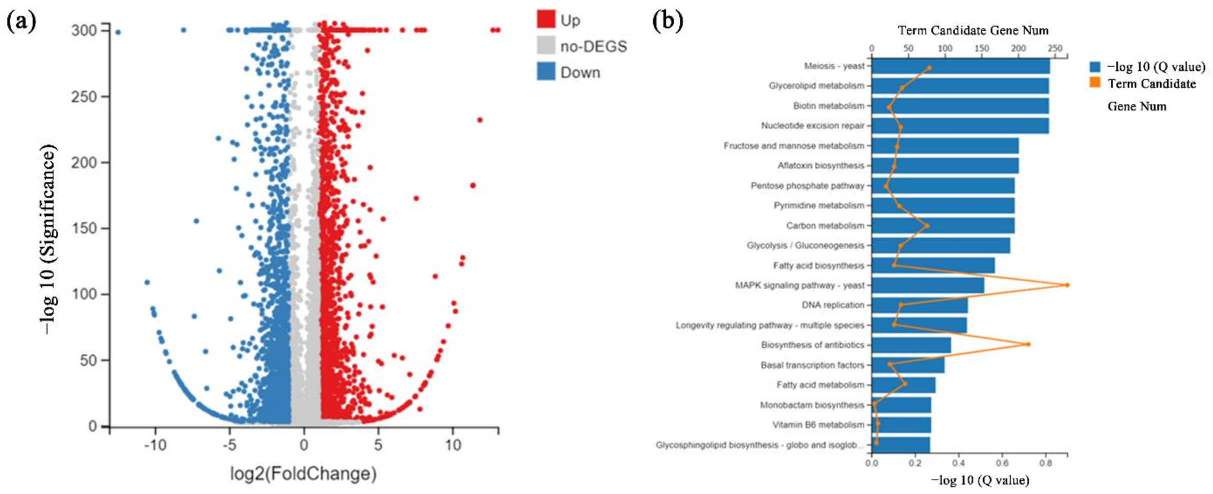

Figure S1 Transcriptome responses and KEGG pathway enrichment analysis. (a) The volcano Fig. of HS + SA/HS group; (b) The top 20 metabolic pathways with significant changes (sorted by Q value from smallest to largest) in the HS + SA vs. HS group.

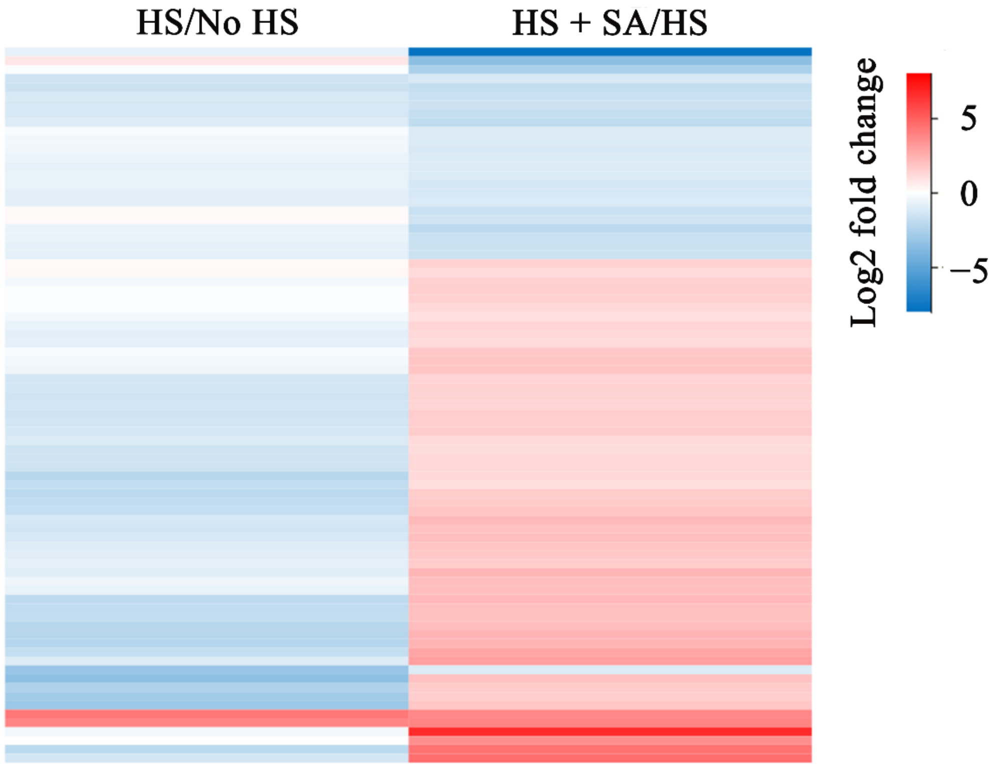

Figure S2 Heat map of changes in gene transcription levels that belonging to "Nucleotide excision repair and DNA replication" in HS/No HS and HS + SA/HS groups.

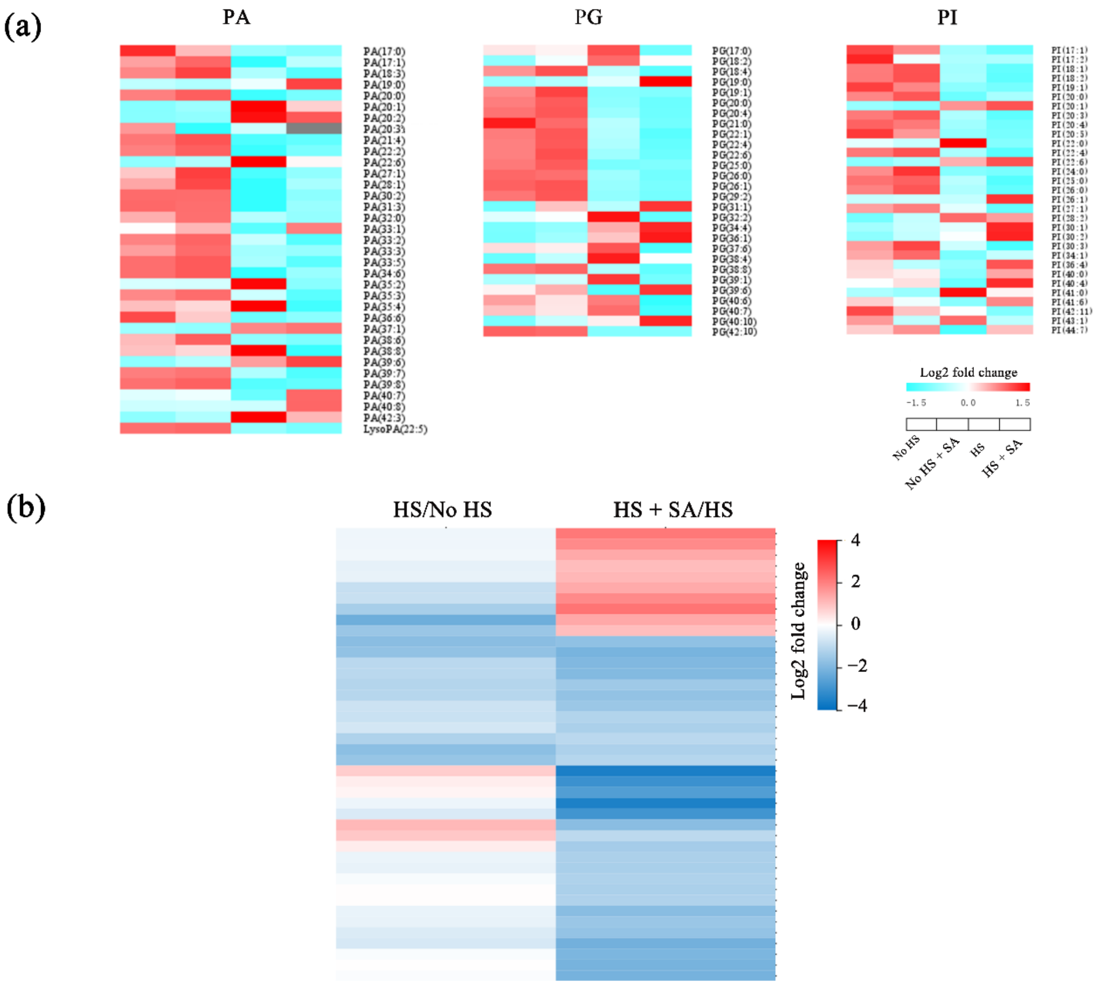

Figure S3 Heat maps of phosphoglycerolipids composition (a) and transcription changes of genes belonging to “Glycerolipid metabolism”(b). PA: phosphatidic acid; PG: phosphatidylglycerol; PI: phosphatidylinositol.

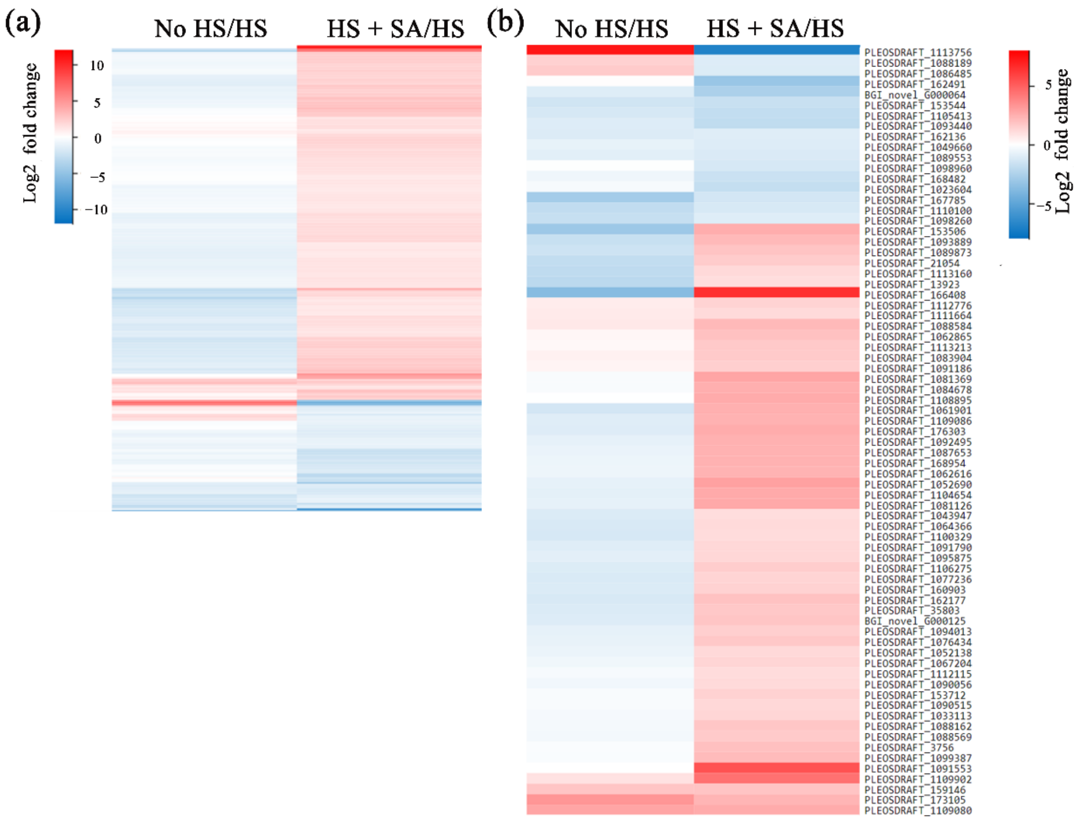

Figure S4 Heat maps of MAPK signaling pathway related genes. (a) Transcription changes of genes belonging to "MAPK signaling pathway". (b) Transcription changes of genes belonging to "cell wall stress pathway" in "MAPK signaling pathway".

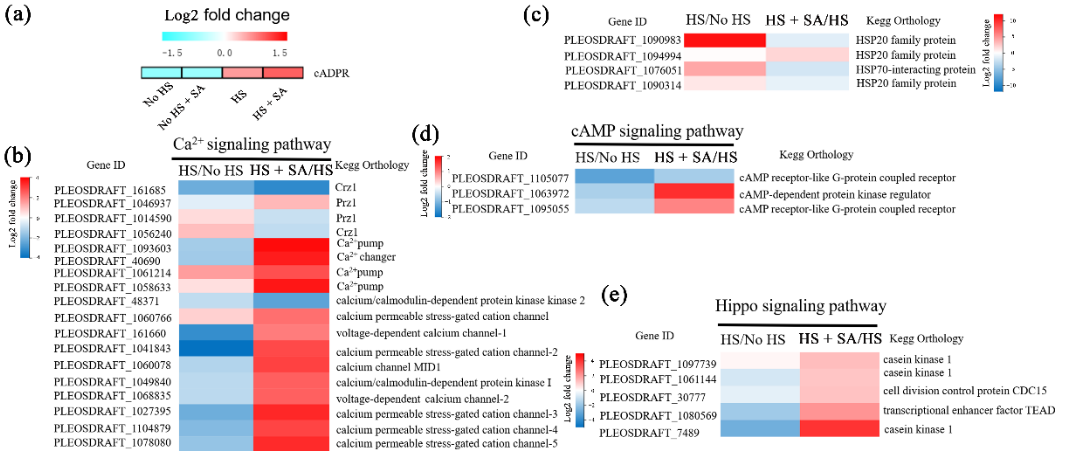

Figure S5 Other signaling pathway analysis. (a) Changes in cADPR metabolism. (b) Transcription changes of genes belonging to "Ca^{2+} signalling pathway". (c) Transcription changes of genes belonging to "heat shock protein family". (d) Transcription changes of genes belonging to "cAMP signalling pathway". (e) Transcription changes of genes belonging to "Hippo signalling pathway".
